# Transmission zone plates as analyzers for efficient parallel 2D RIXS-mapping

**DOI:** 10.1038/s41598-017-09052-0

**Published:** 2017-08-18

**Authors:** Felix Marschall, Zhong Yin, Jens Rehanek, Martin Beye, Florian Döring, Katharina Kubiček, Dirk Raiser, Sreevidya Thekku Veedu, Jens Buck, André Rothkirch, Benedikt Rösner, Vitaliy A. Guzenko, Jens Viefhaus, Christian David, Simone Techert

**Affiliations:** 1 Paul Scherrer Institut, Villigen-PSI, 5232 Switzerland; 2 Deutsches Elektronen-Synchrotron DESY, Photon Science, Hamburg, 22607 Germany; 3 Max Planck Institute for Biophysical Chemistry, Structural Dynamics of (Bio)chemical Systems, Göttingen, 37077 Germany; 4 Georg-August Universität Göttingen, Institute of X-ray Physics, Göttingen, 37077 Germany

## Abstract

We have implemented and successfully tested an off-axis transmission Fresnel zone plate as spectral analyzer for resonant inelastic X-ray scattering (RIXS). The imaging capabilities of zone plates allow for advanced two-dimensional (2D) mapping applications. By varying the photon energy along a line focus on the sample, we were able to simultaneously record the emission spectra over a range of excitation energies. Moreover, by scanning a line focus across the sample in one dimension, we efficiently recorded RIXS spectra spatially resolved in 2D, increasing the throughput by two orders of magnitude. The presented scheme opens up a variety of novel measurements and efficient, ultra-fast time resolved investigations at X-ray Free-Electron Laser sources.

## Introduction

Resonant inelastic X-ray scattering (RIXS) in the soft X-ray regime is a rapidly developing spectroscopic technique with the capability to study elementary excitations of electrons, phonons and spins^1–4^. Especially in the soft X-ray regime, it becomes particularly sensitive to electronic properties of matter^5, 6^ as soft X-ray resonances are the energetically sharpest transitions. Therefore, soft X-rays provide chemical contrast, and can access light elements like C, N and O of chemical relevance. Using RIXS, the band structure of solids^7–9^ as well as the electronic structure of matter in the liquid^10–17^ and the gas phase^18^ has been studied with great success.

Due to the small fraction of inelastically scattered radiation that can be collected, RIXS is often called a photon-hungry analytical technique, and acquisition times are rather long. However, the remarkable progress of X-ray sources in terms of brightness, complemented by substantially improved photon detection instrumentation^19^, opens up new opportunities. In particular one can conceive RIXS setups where additional information is gathered simultaneously with the emission spectrum, for instance to perform RIXS analysis in a spatially or temporally resolved manner^20, 21^. In this context, we present an off-axis transmission Fresnel zone plate (FZP) as advanced 2D RIXS analyzer.

**Figure 1:**
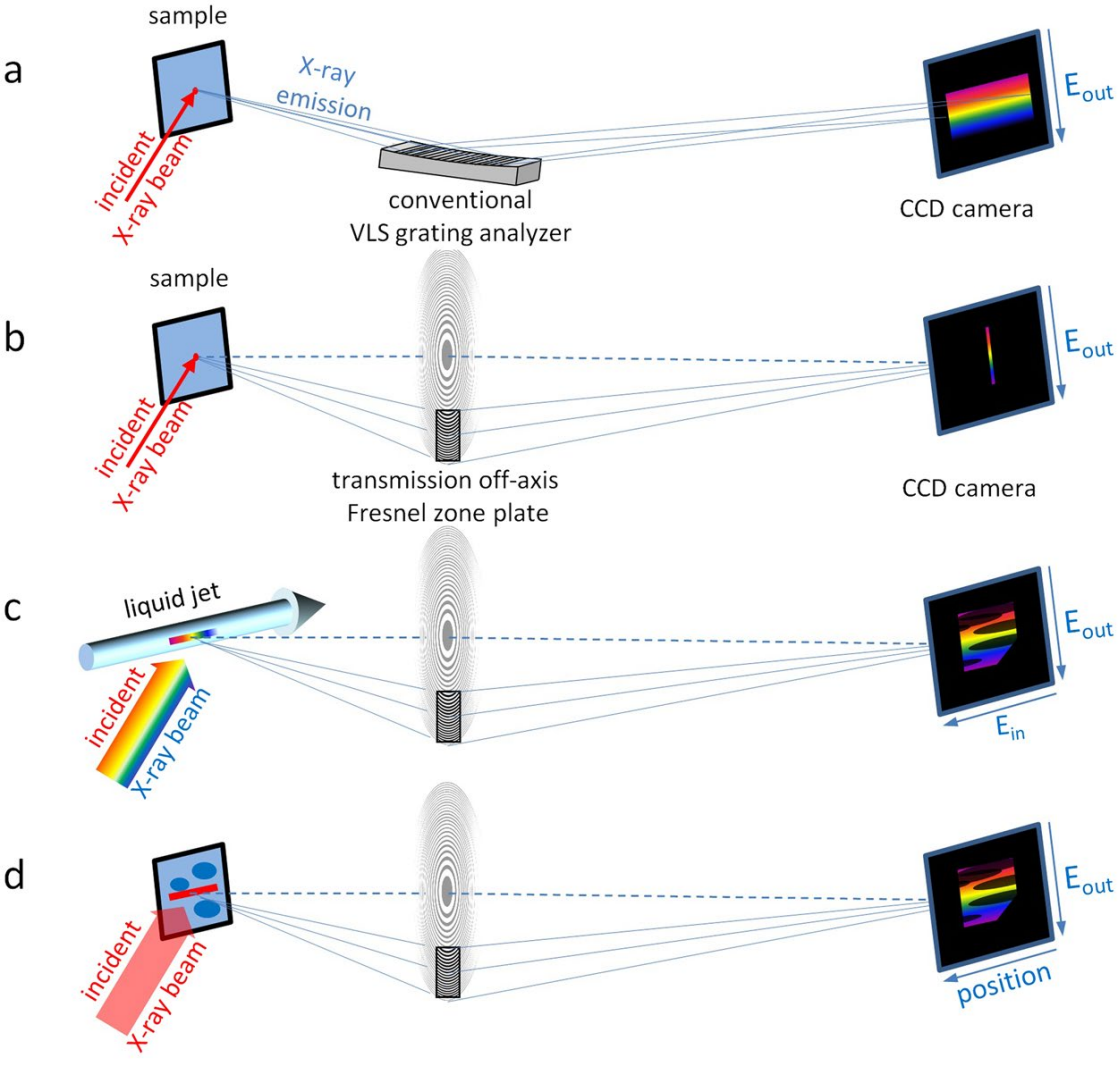
RIXS setup using (**a**) a conventional variable line spacing (VLS) grating analyzer and (**b**)–(**d**) RIXS setups using an off-axis transmission Fresnel zone plate: (**b**) Focused spot on the sample, leading to a fine dispersed line on detector, the direction perpendicular to the dispersion contains no additional information. (**c**) The photon energy varies along a line focus on the sample (e.g. a liquid jet), which enables to record a full RIXS map simultaneously. (**d**) Monochromatic line focus to scan across the sample, whereas each position leads to a spatially resolved RIXS image.

Current typical RIXS analyzers are based on reflecting even line spacing or variable line spacing (VLS) gratings, which collect the emitted light and disperse it across a 2D detector^19, 22–32^. In such an implementation, no information is conveyed along the non-dispersive direction (*E*_out_) of the detector (see Fig. 1a), and the dispersed signal is simply integrated (projected) along this direction. Several concepts have been proposed to exploit the non-dispersive direction on the detector to acquire additional information. These include the scheme of parallel RIXS mapping, also referred to as h*ν* ^2^ -spectroscopy, where the non-dispersive direction on the detector is used to simultaneously collect RIXS spectra for a bandwidth of incident energies^33, 34^. Experimental implementations of such schemes are very rare. Spherical VLS grating analyzers have no imaging capability. Analyzers based on Wolter-type I mirror systems have been implemented^35^, or are under construction^36^. However, no experimental demonstration of parallel RIXS mapping (h*ν* ^2^ -spectroscopy) has been reported to date.

Besides Wolter-type optics, off-axis transmission FZPs also provide imaging capabilities. Recently Marschall *et al*.^37^ reported on a successful test using such an off-axis transmission FZP instead of the VLS grating as analyzer in the RIXS-spectrometer of the ADRESS-beamline at the Swiss Light Source. As a FZP provides focusing properties in 2D, the recorded spectrum is not washed out along the non-dispersive direction, but focused to a fine line (see. Fig. 1b). Using an imaging FZP, the second direction is free for acquiring additional information. For instance, it can be used for h*ν* ^2^ -spectroscopy, where the incoming energy is varied along one axis and the outgoing energy along the other axis (see. Fig. 1c).

## Experimental setup

Here, we present the results from our soft X-ray RIXS spectroscopy measurements using an off-axis transmission Fresnel zone plate spectrometer setup. The experiments were performed at P04 beam line of the PETRA III synchrotron in Hamburg, Germany^38^, utilizing the ChemRIXS end station^39^. The off-axis FZP (3 mm × 3 mm) had 70 nm outermost line period (14285 lines/mm) and 7.53 mm outermost radius. The 370 nm high FZP structures consisted of SiO_2_, supported by a 250 nm thick Si_3_N_4_ -membrane. They were fabricated by exposure of an HSQ (Hydrogen silsesquioxane) resist layer by electron-beam lithography. The FZP was mounted to be energy-dispersive in the horizontal direction.

The FZP had a focal length of 170 mm at 400 eV photon energy and was placed at a distance of 200 mm from the sample to focus onto a CCD-detector at 1.13 m distance from the FZP, which results in a magnification of 5.6. Unlike reflecting VLS analyzer gratings, the transmission zone plates are largely insensitive to tilt, therefore alignment of our transmission FZP took only a few minutes.

As detector we used an ANDOR iKon-M 934 series camera with 1024 × 1024 pixels and a pixel size of 13 μm × 13 μm. The smallest achieved signal spot size on the detector was 6 pixel (FWHM) in the dispersive direction and the dispersion of the spectrometer was 134 meV per pixel. Thus, the best achievable resolution was 804 meV at 400 eV (resolving power *E*/d*E* ~ 500), which is in good agreement with the predicted resolution calculated using the equations shown in a recent publication^37^.

To produce a line focus of 10 μm(h) × 1000 μm(v) on the sample, the vertical monochromator exit slits were fully opened. The mean photon energy was 399.4 eV and varies along the vertical line focus by about 0.7%, roughly a factor of two smaller than the FWHM bandwidth emitted by the 72 period undulator. The fact that we can accept such a wide bandwidth increases the incident flux at least by an order of magnitude compared to the narrowband flux required by a conventional RIXS configuration.

## RIXS mapping

**Figure 2:**
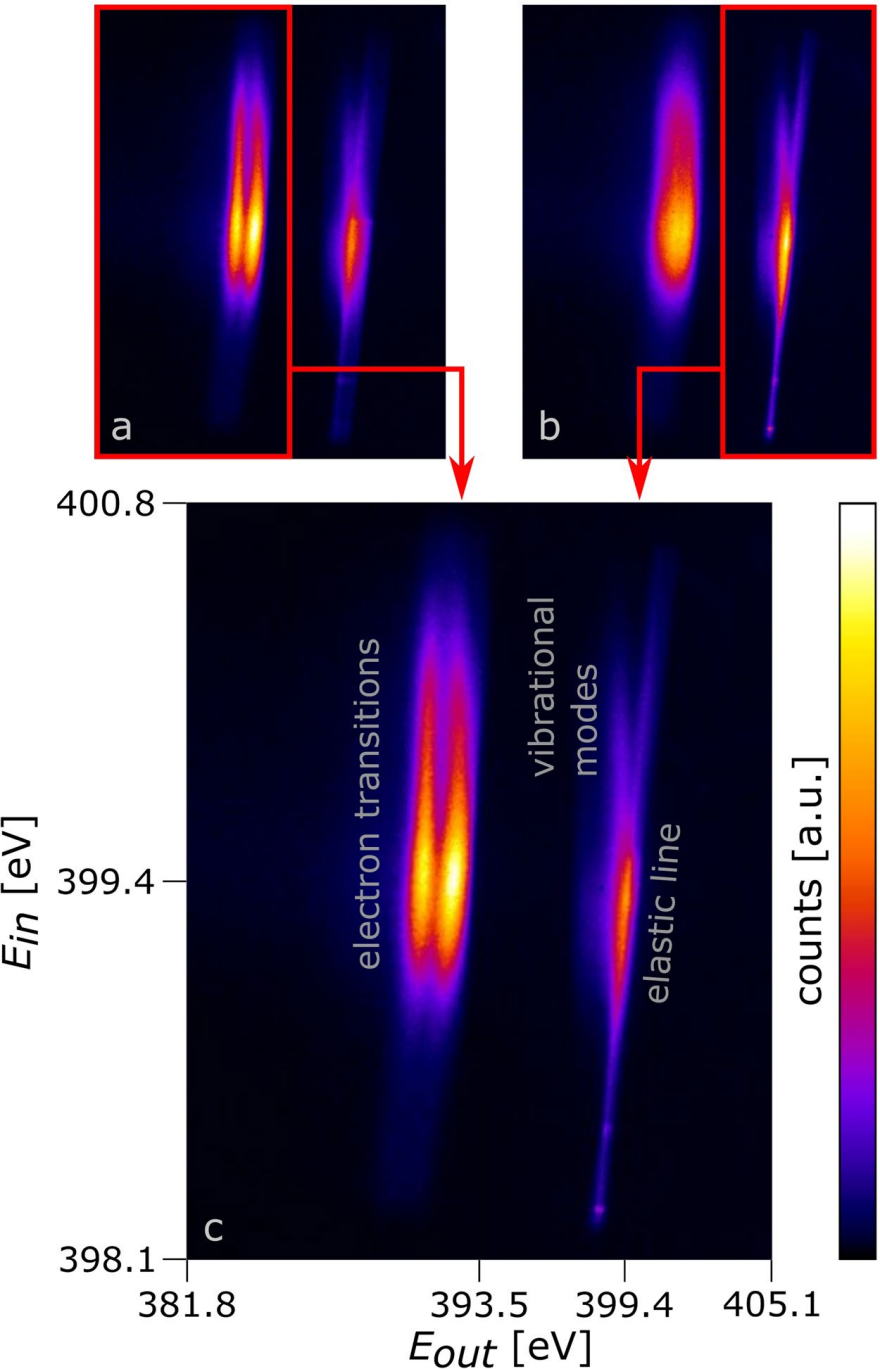
RIXS maps of acetonitrile in a liquid jet experiment at P04, PETRA III, recorded using an off-axis transmission FZP, incident energy 398.2 eV–400.6 eV (elastic line), focus size on liquid jet 10 μm × 1000 μm (h × v). Image (**c**) is a combination of image (**a**), taken at 1.00 m detector distance and image (**b)**, taken at 1.12 m detector distance. (**a**) and (**b**) are the sum of 4 exposures with 240 s exposure time each. Between the exposures, the position of the line focus with respect to the liquid jet was moved in steps of 10 μm to account for slight angular misalignments between the line focus and the jet.

In our experiment we used a round liquid jet with a diameter of ~20 μm of an acetonitrile-ethanol mixture as sample. In the acquired signal shown in Fig. 2, the incident energy is varied along the vertical axis and the outgoing energy is varied along the horizontal axis. As the incoming energy is varied along the line focus, the elastic line is oriented in diagonal direction.

The focal length of the FZP changes with the photon energy. Thus, one image is only focused for one specific energy. We recorded two images, which were acquired at slightly different detector positions, one focused at the elastic line at ~399.4 eV (see Fig. 2b) and the other at the nitrogen emission lines of 7a_1_ and 2e transitions^17^ at ~392 eV (see Fig. 2a). The combination of both images is shown in Fig. 2c. By tilting the detector with respect to the optical axis according to the image positions of the different photon energies, this effect can be partially compensated in the future and it will be possible to obtain a good overall resolution by one exposure only.

**Figure 3:**
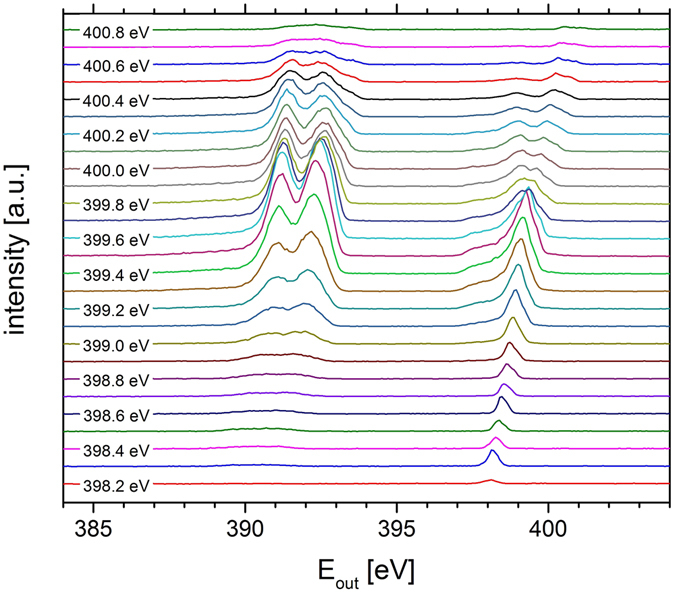
Emission lines of acetonitrile at different excitation energies extracted from the RIXS-map shown in Fig. 2c.

We extracted horizontal intensity profiles from Fig. 2c, resulting in the emission lines shown in Fig. 3. In both images one can identify the elastic line, the electronic transitions and the vibrational modes. The results are in excellent agreement with recent measurements^17, 39, 40^.

The major advantage of our method based on an off-axis transmission FZP is, that entire RIXS maps can be acquired with single images. Taking the spectra shown in Fig. 3 with a conventional RIXS setup would require 27 individual measurements.

## Imaging RIXS

The imaging capabilities of the zone plate analyzer can be also used to efficiently collect spatially resolved RIXS spectra (see. Fig. 1d). RIXS using a conventional analyzer requires illuminating the sample with a tight point focus, while raster-scanning across the sample surface and acquiring RIXS spectra at each point. In our experiment, the sample is illuminated by a line focus and a scan is performed only in the dimension perpendicular to the focus line. In our experiment the line focus had a length of 1000 μm and a width of 10 μm. Therefore scanning the sample with a line focus increases the throughput by two orders of magnitude compared to a 2D scan with a 10 μm × 10 μm spot in a conventional RIXS geometry.

The sample consisted of a silicon wafer coated with 250 nm Si_3_N_4_. On top of the Si_3_N_4_ layer, we patterned 20 nm thick Au structures forming Siemens stars with 500 m diameter and “RIXS” letters with 250 m height. The sample was tilted by 60deg with respect to the incident beam and 30deg with respect to the optical axis of the spectrometer.

**Figure 4:**
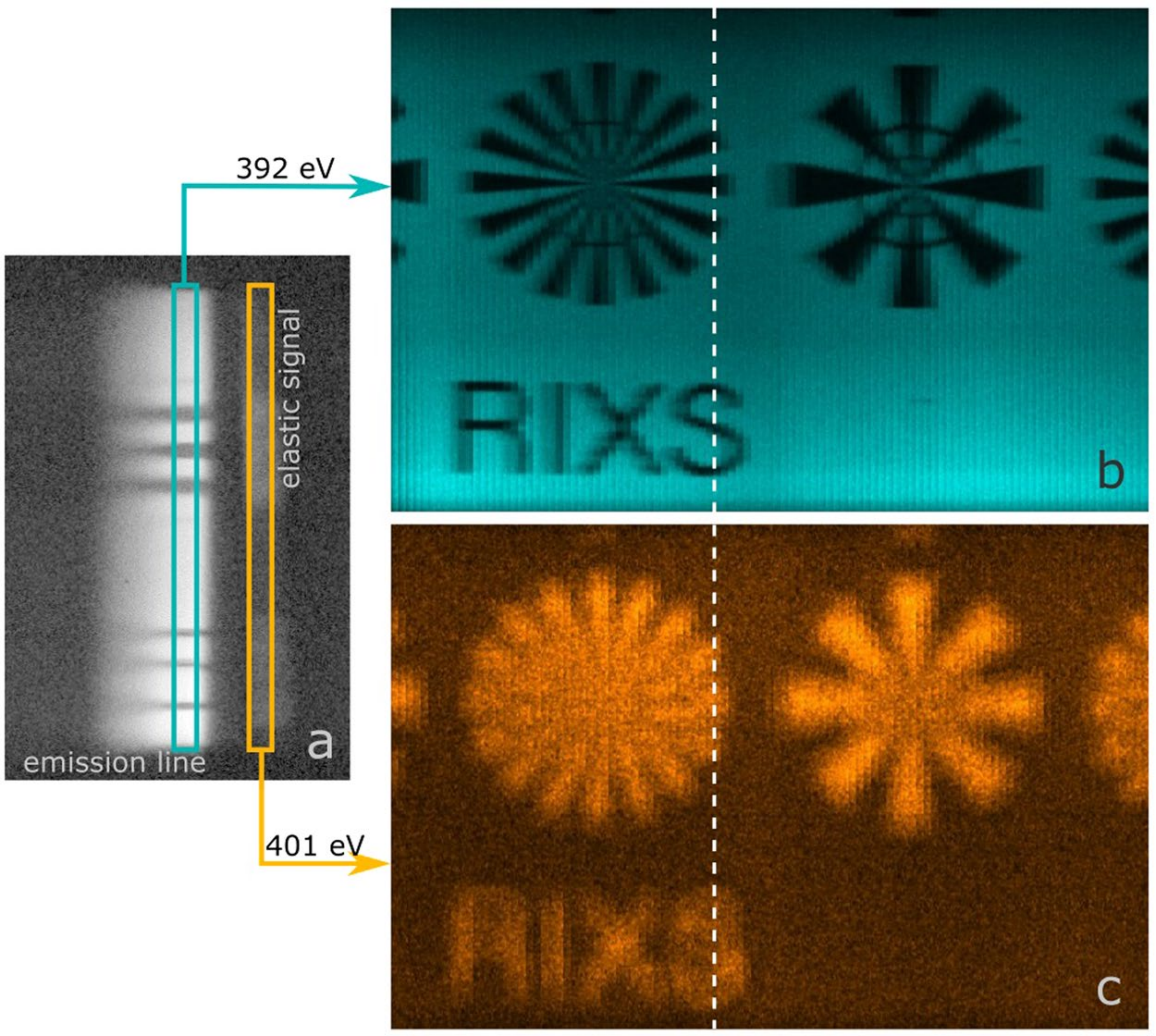
(**a**) RIXS signal of a test sample illuminated with a line focus of 10 μm(h) × 1000 μm(v) at a mean incident photon energy of 401 eV ± 0.35% (elastic peak of Si_3_N_4_) and an exposure time of 20s. (**b**) and (**c**) are reconstructions out of 101 such images, whereby (**b**) is reconstructed from the inelastic Si_3_N_4_ X-ray emission signal and (**c**) from the elastic signal. The dashed line in (**b**) and (**c**) indicates the position of the signal (**a**). The sample was a silicon wafer coated with 250 nm Si_3_N_4_ and 20 nm thick Au patterns. The diameter of the Siemens star was 500 μm. In the left star with the finer spokes, the outer ring structure marks structure width of 25 μm whereas the inner ring marks structure width of 10 μm.

The sample surface was scanned horizontally in 101 steps at a mean incident energy of 401 eV. For each step, the signal of the CCD-detector (Fig. 4a) was recorded. The outgoing energy increases along the horizontal axis from the left to the right. One can identify the electronic transitions, the elastically scattered signal, as well as the band-gap from Si_3_N_4_ in between. From the series of RIXS patterns, we reconstructed an image of the sample from the resonant nitrogen emission at around 392eV (see. Fig. 4b) as well as from the elastically scattered signal at around 401 eV (see Fig. 4c).

In the two-dimensional image displaying emission energy and one spatial dimension at the same time (Fig. 4a), we now use the different emission characteristics of the structured sample to derive two complementary contrast mechanisms that appear with inverted contrast to each other: For an incident energy of 401 eV, Au provides stronger elastic scattering than the Si_3_N_4_ layer. Au appears bright, when selecting 401 eV emission energy, while the regions that lack the Au layer appear darker (Fig. 4b). At the same incident energy, the nitrogen atoms in Si_3_N_4_ are resonantly excited and provide a strong inelastic emission signal at photon energies around 392 eV. Selecting this emission energy, the regions not covered with Au thus show up bright, while the Au cover reduces the nitrogen excitation and inelastic emission, leading to ~80% lower signals at this emission energy (Fig. 4c).

In the inelastically scattered image (Fig. 4b) the spatial resolution is about 10 m horizontally. It is limited by the horizontal size of the spot and the scanning step size. In the vertical direction the spatial resolution is 5 m vertically, being only limited by the imaging properties of the spectrometer as a result of magnification and detector pixel size. Here, with a magnification of 5.6 and detector pixels of 13.5 μm, 2-fold sampling leads to a theoretical resolution limit of 4.6 μm. The intrinsic diffraction limit of the FZP depending on the numerical aperture *NA* and the wavelength *λ* is *λ*/*NA* ~ 400 nm and thus not limiting the resolution here.

In the image reconstructed form the elastic line (Fig. 4c), the spatial resolution is worse, due to the chromatic aberration, as the system was focused for the inelastic emission signal at 392 eV.

## Discussion

The opportunity to collect spatial and electronic information simultaneously is highly interesting as it enables to efficiently record local changes of the sample’s electronic states^41^. These can be caused by artificial structures like in our experiment, but one can also investigate the local changes of the native characteristics of the material itself, revealing the materials properties at defects and interfaces. In the future the spatial resolution as well as the energy resolution can be increased by a reduction of the horizontal spot size on the sample and by a reduction of the effective detector pixel size.

Due to the chromatic aberration of FZPs, their focal length is changing linearly with the photon energy. As the depth of field is limited, the spectrometer can only be perfectly focused for one photon energy at the same time. We compensated for this effect by taking two images at different detector positions. This effect can likely be reduced by tilting the detector in future experiments. Tilting the detector would also reduce the effective pixel size in the dispersive direction leading to increasing energy resolution.

In summary, the use of an off-axis transmission FZP for RIXS enables exploiting the second axis of the detector order to obtain additional information, which opens up a variety of possible applications. In our experiments, we varied the incident energy across a homogeneous sample as well as the local material properties of a microstructured sample. In the future one could also vary properties like time, temperature, chemical composition, and others on the second axis, making this RIXS analyzer scheme very versatile.

## References

[1] Bisogni V (2016). Ground-state oxygen holes and the metal-insulator transition in the negative charge-transfer rare-earth nickelates. Nature Communications.

[2] Johnston S (2016). Electron-lattice interactions strongly renormalize the charge-transfer energy in the spin-chain cuprate Li2CuO2. Nature Communications.

[3] Zhou K-J (2013). Persistent high-energy spin excitations in iron-pnictide superconductors. Nature Communications.

[4] Ament LJP, van Veenendaal M, Devereaux TP, Hill JP, van den Brink J (2011). Resonant inelastic X-ray scattering studies of elementary excitations. Reviews of Modern Physics.

[5] Kotani A, Shin S (2001). Resonant inelastic X-ray scattering spectra for electrons in solids. Reviews of Modern Physics.

[6] Himpsel FJ (2011). Photon-in photon-out soft X-ray spectroscopy for materials science. physica status solidi (b).

[7] Ma Y (1992). Soft-X-ray resonant inelastic scattering at the C K edge of diamond. Physical Review Letters.

[8] Beye M, Sorgenfrei F, Schlotter WF, Wurth W, Fohlisch A (2010). The liquid-liquid phase transition in silicon revealed by snapshots of valence electrons. Proceedings of the National Academy of Sciences.

[9] Peng, Y. Y. *et al*. Magnetic excitations and phonons simultaneously studied by resonant inelastic X-ray scattering in optimally doped Bi1.5Pb0.55Sr1.6La0.4CuO6+*δ*. *Physical Review B* **92** (2015).

[10] Fuchs, O. *et al*. Isotope and temperature effects in liquid water probed by X-ray absorption and resonant x-ray emission spectroscopy. *Physical Review Letters* **100** (2008). 10.1103/PhysRevLett.100.027801 18232928

[11] Weinhardt L (2013). RIXS investigations of liquids, solutions, and liquid/solid interfaces. Journal of Electron Spectroscopy and Related Phenomena.

[12] Wernet P (2009). Structure and dynamics in liquid water from X-ray absorption spectroscopy. Journal of Physics: Conference Series.

[13] Yin, Z. *et al*. Ionic solutions probed by resonant inelastic X-ray scattering. *Zeitschrift für Physikalische Chemie* **229** (2015).

[14] Yin Z (2014). Probing the hofmeister effect with ultrafast core-hole spectroscopy. The Journal of Physical Chemistry B.

[15] Wernet P (2015). Orbital-specific mapping of the ligand exchange dynamics of Fe(CO)5 in solution. Nature.

[16] Harada, Y. *et al*. Selective probing of the OH or OD stretch vibration in liquid water using resonant inelastic soft-X-ray scattering. *Physical Review Letters* **111** (2013). 10.1103/PhysRevLett.111.193001 24266469

[17] Dierker B (2013). Probing orbital symmetry in solution: polarization-dependent resonant inelastic soft X-ray scattering on liquid micro-jet. New Journal of Physics.

[18] Hennies, F. *et al*. Resonant inelastic scattering spectra of free molecules with vibrational resolution. *Physical Review Letters* **104** (2010). 10.1103/PhysRevLett.104.193002 20866962

[19] Bilderback DH, Elleaume P, Weckert E (2005). Review of third and next generation synchrotron light sources. Journal of Physics B: Atomic, Molecular and Optical Physics.

[20] Salek P, Baev A, Gel’mukhanov F, Agren H (2003). Dynamical properties of X-ray raman scattering. Phys. Chem. Chem. Phys..

[21] Rubensson J-E (2000). RIXS dynamics for beginners. Journal of Electron Spectroscopy and Related Phenomena.

[22] Ghiringhelli G (2006). SAXES, a high resolution spectrometer for resonant X-ray emission in the 400–1600ev energy range. Review of Scientific Instruments.

[23] Chiuzbaian SG (2014). Design and performance of AERHA, a high acceptance high resolution soft X-ray spectrometer. Review of Scientific Instruments.

[24] Dallera C (1996). Soft X-ray emission spectroscopy at ESRF beamline 26 based on a helical undulator. Journal of Synchrotron Radiation.

[25] Harada Y (2012). Ultrahigh resolution soft X-ray emission spectrometer at BL07LSU in SPring-8. Review of Scientific Instruments.

[26] Poletto L (2014). Spectrometer for x-ray emission experiments at FERMI free-electron-laser. Review of Scientific Instruments.

[27] Chuang Y-D (2017). Modular soft X-ray spectrometer for applications in energy sciences and quantum materials. Review of Scientific Instruments.

[28] Tanikawa T (2016). First observation of SASE radiation using the compact wide-spectral-range XUV spectrometer at FLASH2. Nuclear Instruments and Methods in Physics Research Section A: Accelerators, Spectrometers, Detectors and Associated Equipment.

[29] Nordgren J (1989). Soft X-ray emission spectroscopy using monochromatized synchrotron radiation (invited). Review of Scientific Instruments.

[30] Callcott TA, Tsang KL, Zhang CH, Ederer DL, Arakawa ET (1986). High-efficiency soft X-ray emission spectrometer for use with synchrotron radiation excitation. Review of Scientific Instruments.

[31] Yin Z (2015). A new compact soft X-ray spectrometer for resonant inelastic X-ray scattering studies at PETRA III. Review of Scientific Instruments.

[32] Qiao Rea (2017). Advanced light source high-efficiency *in situ* resonant inelastic X-ray scattering (iRIXS) endstation at the advanced light source. Rev. Sci. Instrum..

[33] Strocov VN (2009). Concept of a spectrometer for resonant inelastic X-ray scattering with parallel detection in incoming and outgoing photon energies. Journal of Synchrotron Radiation.

[34] Rehanek J (2013). A case study of novel X-ray optics for FEL sources. *Journal of Physics*. Conference Series.

[35] Yamane H, Kosugi N, Hatsui T (2013). Transmission-grating spectrometer for highly efficient and high-resolution soft X-ray emission studies. Journal of Electron Spectroscopy and Related Phenomena.

[36] Warwick T, Chuang Y-D, Voronov DL, Padmore HA (2014). A multiplexed high-resolution imaging spectrometer for resonant inelastic soft X-ray scattering spectroscopy. Journal of Synchrotron Radiation.

[37] Marschall F (2017). Zone plates as imaging analyzers for resonant inelastic X-ray scattering. Opt. Express.

[38] Viefhaus J (2013). The variable polarization XUV beamline P04 at PETRA III: Optics, mechanics and their performance. Nuclear Instruments and Methods in Physics Research Section A: Accelerators, Spectrometers, Detectors and Associated Equipment.

[39] Yin Z (2017). An endstation for resonant inelastic X-ray scattering studies of solid and liquid samples. Journal of Synchrotron Radiation.

[40] Niskanen J (2016). Density functional simulation of resonant inelastic X-ray scattering experiments in liquids: acetonitrile. Phys. Chem. Chem. Phys..

[41] Campi G (2015). Inhomogeneity of charge-density-wave order and quenched disorder in a high-Tc superconductor. Nature.

